# The association between dietary amino acid profile and the risk of type 2 diabetes: Ravansar non-communicable disease cohort study

**DOI:** 10.1186/s12889-023-17210-5

**Published:** 2023-11-18

**Authors:** Farid Najafi, Parisa Mohseni, Yahya Pasdar, Mahdieh Niknam, Neda Izadi

**Affiliations:** 1 Research Center for Environmental Determinants of Health (RCEDH), Health Institute, Kermanshah University of Medical Sciences, Kermanshah, Iran; 2 Department of Epidemiology, School of Public Health and Safety, Shahid Beheshti University of Medical Sciences, Tehran, Iran; 3 Research Center for Social Determinants of Health, Research Institute for Endocrine Sciences, Shahid Beheshti University of Medical Sciences, Tehran, Iran

**Keywords:** Dietary amino acid, FFQ, Type 2 Diabetes, PERSIAN Cohort

## Abstract

**Background:**

Type 2 diabetes (T2D) is one of the most common chronic diseases and the main risk factors for T2D consist of a combination of lifestyle, unhealthy diet, and genetic factors. Amino acids are considered to be a major component of dietary sources for many of the associations between dietary protein and chronic disease. Therefore, this study amied to determine the association between dietary amino acid intakes and the incidence of T2D.

**Methods:**

The present nested case-control study was conducted using data from the Ravansar Non-Communicable Disease (RaNCD) Cohort Study. The information required for this study was collected from individuals who participated in the Adult Cohort Study from the start of the study until September 2023. Over a 6-year follow-up period, data from 113 new T2D cases were available. Four controls were then randomly selected for each case using density sampling. Cases and controls were matched for sex and age at the interview. Food frequency questionnaire (FFQ) was used to collect data related to all amino acids including tryptophan, threonine, isoleucine, leucine, lysine, methionine, cysteine, phenylalanine, tyrosine, valine, arginine, histidine, alanine, aspartic acid, glutamic acid, glycine, proline, and serine were also extracted. Binary logistic regression was used to estimate the crude and adjusted odds ratio for the risk of T2D.

**Results:**

Using the univariable model, a significant association was found between T2D risk and branched-chain, alkaline, sulfuric, and essential amino acids in the fourth quartile. Accordingly, individuals in the fourth quartile had a 1.81- to 1.87-fold higher risk of developing new T2D than individuals in the lowest quartile (*P*<0.05). After adjustment for several variables, the risk of developing a new T2D was 2.70 (95% CI: 1.16-6.31), 2.68 (95% CI: 1.16-6.21), 2.98 (95% CI: 1.27-6.96), 2.45 (95% CI: 1.02-5.90), and 2.66 (95% CI: 1.13-6.25) times higher, for individuals in the fourth quartile of branched-chain, alkaline, sulfuric, alcoholic, and essential amino acids compared with those in the lowest quartile, respectively.

**Conclusions:**

The results showed that the risk of developing a new T2D was higher for individuals in the fourth quartile of branched-chain amino acids, alkaline, sulfate, and essential amino acids than in the lower quartile.

**Supplementary Information:**

The online version contains supplementary material available at 10.1186/s12889-023-17210-5.

## Background

Type 2 diabetes (T2D) is one of the most common chronic diseases and is strongly related to cardiovascular disease (CVD), hypertension, and certain types of cancer [1]. Previous studies have consistently demonstrated that a combination of lifestyle, diet, and genetic factors could affect the risk of developing T2D [2]. Amino acids are also considered to be a major component of dietary sources for many of the previously reported associations between dietary protein and chronic disease [1, 3, 4]. There is evidence that branched-chain and aromatic amino acids are associated with higher T2D risk, but there is conflicting evidence regarding other amino acids [5, 6]. Findings from a meta-analysis of eight prospective studies illustrated that the higher dietary intake of amino acids including isoleucine, leucine, valine, tyrosine, and phenylalanine was associated with a higher risk of T2D. Results from a previous study demonstrated an inverse association between glycine and glutamine and the risk of developing T2D [7]. In addition, the results of two studies in Germany and Finland showed an inverse association between glycine and the occurrence of T2D, whereas no association was observed in South Asians and immigrants in the United Kingdom [8–10]. Furthermore, whereas some studies revealed an inverse association between glutamine and T2D risk [8, 11, 12], this association was not significant in other related studies [9, 10]. While studies rarey investigated the relationship between histidine and alanine amino acids and risk of T2D, one study showed a positive association [7].

Most studies have been conducted in European and American populations, whereas this association has been rarely investigated in Asian populations and conflicting results emerged from these studies. In China, a prospective study examined three branched-chain amino acids (BCAA) and two aromatic amino acids in relation to insulin resistance and the development of T2D and highlighted the predictive value of these markers for the development of T2D [13]. In this regard, a prior study found a positive association between combined scores of nineteen amino acids and T2D among the Japanese adult population [12]. In addition, the study by Chen et al. (2019) showed that the intake of alanine, valine, leucine, tyrosine, isoleucine, phenylalanine, lysine, glutamate, and ornithine significantly contributed to the occurrence of T2D [14]. These diverse findings may suggest the ethnic-specific differences in the association between different amino acid intakes and T2D in Western and Asian countries [9]. Given the ethnic differences between Asian countries in the interaction between genetic, pathophysiological, cultural, and lifestyle factors affecting T2D [15], it expects to see a specific pattern of interaction between amino acids and diabetes among Asian populations and in countries such as Iran. As dietary amino acid patterns could related to T2D in different ways, this study aimed to determine the association between dietary amino acid intakes and the incidence of type 2 diabetes in the adult population participating in Ravansar Non-Communicable Disease (RaNCD) Cohort Study.

## Methods

### Study population

**Fig. 1:**
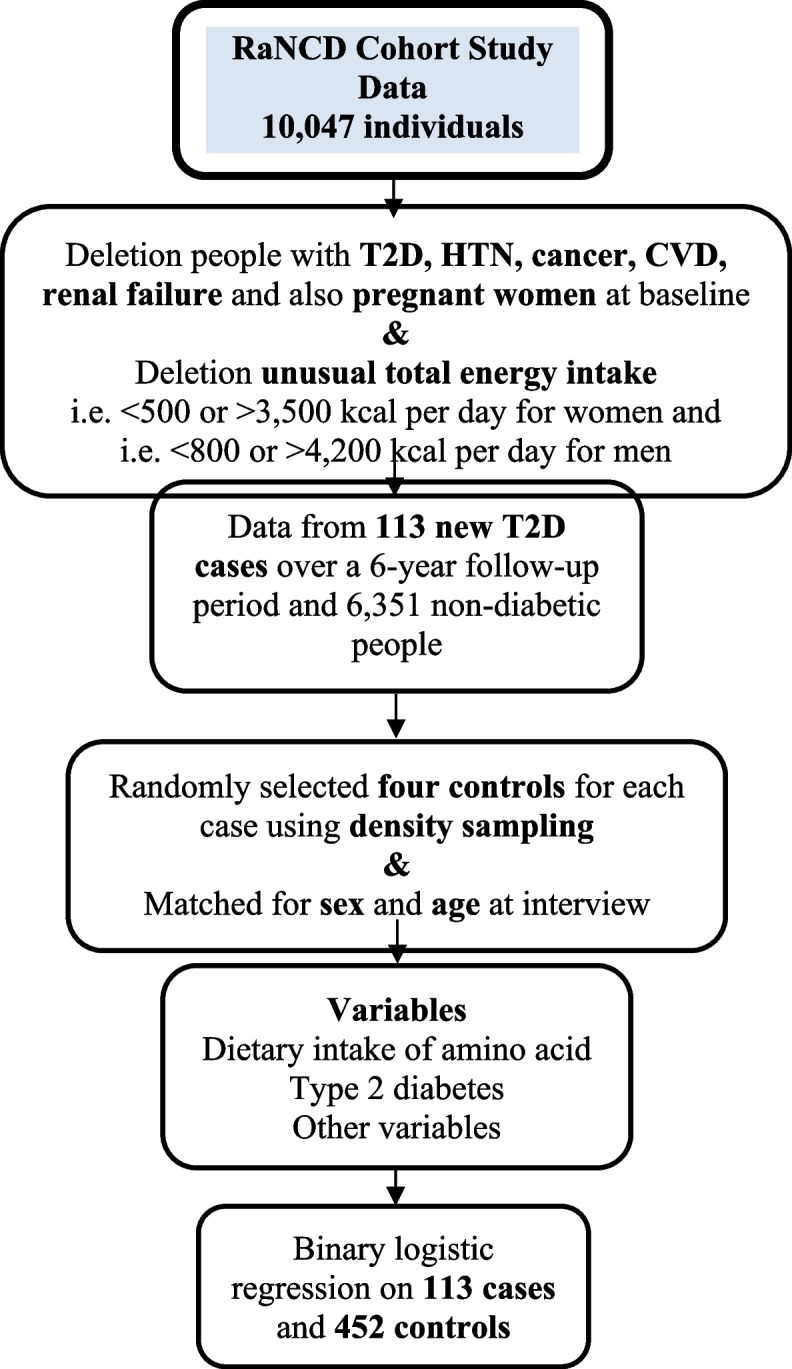
Flowchart of the study participants and data preparation

The present nested case-control study was conducted using data from the Ravansar Non-Communicable Disease (RaNCD) Cohort Study. The RaNCD cohort study is part of the PERSIAN (Prospective Epidemiological Research Studies in IrAN) Cohort and is a population-based prospective study of a group of individuals aged 35–65 years at different phases. The sample size of the main group is at least 10,000 individuals. More details about this cohort are available elsewhere [16]. The information required for this study was collected from individuals who participated in the Adult Cohort Study from the start of the study until September 2023. At first, the men and women who were diagnosed with T2D (795), hypertension (HTN) (1,332), cancer (67), cardiovascular disease (1,221), renal failure (4), and also pregnant women (93) at baseline and who had an unusual total energy intake (i.e. <500 or > 3,500 kcal per day for women and i.e. <800 or > 4,200 kcal per day for men) (1,078) [17, 18] were excluded from the data. After exclusion, data from 113 new T2D cases over a 6-year follow-up period were available for the study. Four controls were then randomly selected for each case using density sampling. Cases and controls were individually matched for sex and age at the interview (Fig. 1). All participants gave written informed consent for this study, which was approved by the Kermanshah University of Medical Sciences Review Board.

### Data collection and measurements

#### Dietary intake of amino acid (main exposure)

The national Iranian food frequency questionnaire (FFQ) was used to determine participants’ usual food intake at the time of recruitment. The FFQ consisted of questions about the frequency of consumption of 125 food items and the corresponding standard serving sizes (e.g., glass, cup, slice, teaspoon, tablespoon, spatula, cube, etc.). The validity and reproducibility of a food intake frequency questionnaire in the PERSIAN Cohort Study was assessed in the study by Eghtesad et al. [19]. Participants reported the average frequency and portion size of foods consumed in the past year. To minimize recall bias, the FFQ was administered by trained dietitians, and participants were given sufficient time to recall the consumption of each food. The FFQs were then analyzed using Nutritionist IV software, which is based on U.S. Department of Agriculture Food Composition data (USDA National Nutrient Database for Standard Reference, Release 28, 2015), to determine energy and nutrient intakes. All amino acids including tryptophan, threonine, isoleucine, leucine, lysine, methionine, cysteine, phenylalanine, tyrosine, valine, arginine, histidine, alanine, aspartic acid, glutamic acid, glycine, proline, and serine was converted to grams per day (g/day) to measure the daily intake of each amino acid and then were classified into eight groups based on their chemical structure, including branched-chain (leucine, isoleucine, valine), aromatic (tryptophan, phenylalanine, tyrosine), alkaline (histidine, arginine, lysine), sulfuric (methionine, cysteine), acidic (glutamic acid, aspartic acid), alcoholic (serine, threonine), small amino acids (glycine, alanine), and cyclic side chain (proline). In addition, two groups of essential (histidine, isoleucine, leucine, lysine, methionine, phenylalanine, threonine, tryptophan, valine) and nonessential (alanine, arginine, aspartic acid, cysteine, glutamic acid, glycine, proline, serine, tyrosine) amino acids were added to the model as the main exposure. A quartile was also used for the amino acid groups. A higher quartile indicates an elevated level of dietary amino acid intake, reflecting increased consumption of amino acids.

### Diabetes

Diabetes was identified by a fasting plasma glucose (FPG) ≥ 126 mg/dl dL [7 mmol per L] and/or use of diabetes medications (insulin and/or oral hypoglycemic agents) in individuals who did not have the disease at baseline in the cohort study [20].

To control for confounding factors, variables such as age, gender, education level, smoking status, alcohol consumption, physical activity, socioeconomic status, dietary habits, macronutrients, anthropometric characteristics, underlying diseases (self-reported), sleep habits, etc., were also extracted from the demographic and clinical information section of the Persian cohort questionnaire used in the Ravansar cohort. The details of data collection and measurements have been described in detail elsewhere [20–24].

In brief, participants were classified as current smokers, never smokers, passive smokers, and ex-smokers based on smoking status and intensity of smoking. Physical activity was measured by 24-hour physical activity and a 22-item questionnaire and was classified as low (24-36.5 metabolic equivalent = MET/hours per day), moderate (36.6–44.4 MET/hours per day), and vigorous (≥ 44.5 MET/hours per day) [25]. Socioeconomic status (SES) was defined using asset data. Total asset scores were calculated using a principal component analysis (PCA), which is the sum of the scores for each asset variable. Dietary habits were assessed using a valid and reliable FFQ questionnaire. Also, healthy and unhealthy patterns were calculated using factor analysis. A BIA device (InBody 770 BIOSPACE, Korea) and a BSM 370 (Biospace Co, Seoul, Korea) were used for weight and height measurements (with 0.5 kg and 0.1 cm accuracy, respectively) and body mass index (BMI) were calculated using weight (kg)/height^2^ (m) formula. Subjects were categorized as underweight with a BMI < 18.5 kg/m^2^, normal weight with 18.5 ≤ BMI ≤ 24.9 kg/m^2^, overweight with 25 ≤ BMI ≤ 29.9 kg/m^2^, and obese with a BMI ≥ 30 kg/m^2^ [26]. Waist-to-height ratio (WHtR) was defined as waist circumference (cm) /hip (cm) [24]. Dyslipidemia was defined as total cholesterol of ≥ 240 mg/dl and/or triglycerides of ≥ 200 mg/dl and/or low-density lipoprotein (LDL) cholesterol of ≥ 160 mg/dl and/or high-density lipoprotein (HDL) cholesterol of < 40 mg/dl and/or taking medication for dyslipidemia [27, 28]. For metabolic syndrome, three or more of the following criteria must be met; elevated blood pressure (BP), defined as systolic BP ≥ 130 mmHg and/or diastolic BP ≥ 85 mmHg or medication used to treat hypertension, elevated triglycerides (TG) ≥ 150 mg/dl or medication use to treat hypertriglyceridemia, HDL-C < 50 mg/dl or medication use for low HDL-C, elevated fasting blood glucose (FBS) ≥ 100 or medication use for treatment of diabetes, central obesity (waist circumference (WC) ≥ 91 cm) [29]. Data on thyroid disease and diabetes in the family were based on self-report. In addition, the time between falling asleep and waking up was defined as sleep duration and categorized as < 6 h, 6–8 h, and > 8 h; and the duration the participant was in bed before actually falling asleep was defined as duration of falling asleep (< 15 min or ≥ 15 min).

### Statistical analysis

Mean (standard deviation), median (IQR = interquartile range) (for non-normal distribution), and number (percentage) were used to describe quantitative and qualitative variables. Because intakes of most specific nutrients correlate with total energy intake, a residual adjustment was made for total energy [30–32]. The Chi-square, T-test, and Mann-Whitney test were used to compare the frequency of categorical variables and the distribution of continuous variables between two groups (case and control). Binary logistic regression was used to estimate the crude and adjusted odds ratio (OR) for the risk of T2D [33]. To determine an association between dietary amino acids and T2D risk, all variables with a P-value less than 0.2 in the univariable model were included in the multivariable analysis. Models were adjusted for residency, SES, education level, family history of diabetes, BMI, WHtR, physical activity, sleep duration, dietary patterns, comorbidities, systolic and diastolic blood pressure, and daily energy intake. In addition, the restricted cubic spline method was used to assess the non-linear relationship between dietary amino acid and the risk of T2D. Different models with different knots (nknot = 3–7) and quadratic, and cubic terms of the amino acid profile were fitted to the data. Data were analyzed using Stata (version 15) and R (version 4.2.0) software. For all statistical tests, *P* < 0.05 was considered statistically significant.

## Results

Most people with T2D lived in urban areas (70.80% vs. 53.10%) (*P* = 0.001). The frequency of high SES was significantly lower in diabetics than in the control group (21.24% vs. 27.43%). The frequency of current and former smokers was 15.93% in patients with T2D and 16.70% in the control group. Alcohol consumption was slightly higher in the control group than in the case group (2.88% vs. 2.65%). But there is no difference between the case and control groups based on the findings. The frequency of low physical activity was higher in patients with T2D than in the control group (32.74% vs. 28.98%). Sleep duration of less than 6 h and more than 8 h was significantly higher in the case group than in the control group (*P* = 0.03). The frequency of falling asleep for less than 15 min was also higher in patients with T2D, but this finding was not statistically significant (*P* = 0.27). The frequency of adherence to a healthy dietary pattern was higher in T2D patients than in the control group. The frequency of comorbidities, such as dyslipidemia and metabolic syndrome were significantly higher than in control group (*P* < 0.001). Mean anthropometric indices (BMI, WC, and WHtR) and systolic and diastolic blood pressure were significantly higher in patients with T2D than in non-diabetics (*P* < 0.05). The frequency of family history of diabetes in patients with T2D was significantly higher than in the control group (42.48% vs. 22.79%, respectively) (Table 1). In addition, The amount of food group consumption in the case and control groups is shown in Appendix 1. Based on the results, the median of intake of all amino acids (except for alcholic and proline) was higher in patients with T2D than in the control group, and this result was statistically significant (*P* < 0.05) (Table 2).

**Table 1:** Frequency and distribution of different variables by group (case & control)

Variables	Case (***n***= 113)	Control (***n***= 452)	***P***-value^b^
	N (%)		
**Education (year)**^a^	4.15 (4.29)	4.87 (4.99)	0.16^**b**^
**Residency**			
Urban	80 (70.80)	240 (53.10)	**0.001**
Rural	33 (29.20)	212 (46.90)	
**Socio-economic status**			
Poor	44 (38.94)	191 (42.26)	0.12
Moderate	45 (39.82)	137 (30.31)	
High	24 (21.24)	124 (27.43)	
**Smoking status**			
Non**-**smoker & Passive smoker	95 (84.07)	374 (83.30)	0.89
Current & *Ex-smokers*	18 (15.93)	75 (16.70)	
**Alcohol consumption**			
Yes	3 (2.65)	13 (2.88)	0.89
No	110 (97.35)	439 (97.12)	
**Physical activity (MET h/day)**			
Low	37 (32.74)	131 (28.98)	**0.03**
Moderate	57 (50.44)	233 (51.55)	
High	19 (16.81)	88 (19.47)	
**Anthropometry**^a^			
Body Mass Index, kg/m^2^	30.10 (4.73)	26.79 (4.73)	**<0.001**^**c**^
Waist Circumference, cm	101.59 (9.74)	95.96 (9.97)	**<0.001**^**c**^
Waist-to-height ratio	0.97 (0.06)	0.93 (0.05)	**<0.001**^**c**^
**Sleep duration (per 24 h)**			
<6 h	13 (11.50)	46 (10.18)	**0.02**
6**-**7 h	51 (45.13)	244 (53.98)	
≥8 h	49 (43.36)	162 (35.84)	
**Falling sleep duration**			
<15 min	77 (68.14)	283 (62.61)	0.27
≥15 min	36 (31.86)	169 (37.39)	
**Healthy pattern (dietary)**			
Tertile 1	27 (23.89)	162 (35.84)	**0.04**
Tertile 2	41 (36.28)	147 (32.52)	
Tertile 3	45 (39.82)	143 (31.64)	
**Unhealthy pattern (dietary)**			
Tertile 1	35 (30.97)	154 (34.07)	0.24
Tertile 2	45 (39.82)	143 (31.64)	
Tertile 3	33 (29.20)	155 (34.29)	
**Total energy (Kcal/day)**^a^	2540.77 (687.19)	2422.86 (722.86)	0.11
**Macronutrients**			
Protien (gr/day)	387.43 (112.68)	373.62 (115.87)	0.07
Carbohydrate (gr/day)	86.05 (30.68)	80.43 (28.71)	0.25
Fat (gr/day)	76.23 (26.32)	71.03 (26.10)	0.06
**Comorbidities (yes)**			
Dyslipidemia	60 (53.10)	157 (34.73)	**<0.001**
Metabolic syndrome	62 (54.86)	63 (13.93)	**<0.001**
Thyroid disease	16 (14.16)	43 (9.51)	0.14
**Family history of diabetes (yes)**	48 (42.48)	103 (22.79)	**<0.001**
**Blood pressure**^a^			
Systolic	107.39 (12.59)	102.96 (12.72)	**0.001**^**c**^
Diastolic	69.58 (8.05)	67.**03** (7.80)	**0.002**^**c**^

^a^Mean (Standard deviation); ^b^Based on chi-square test; ^c^Based on t-test

**Table 2:** Distribution of amino acids profile by group

Amino Acids	Case (***n***= 113)	Control (***n***= 452)	***P***-value^**a**^
	Median (IQR) (gr/day)		
**Branched-chain**	8.15 (4.74)	7.78 (5.10)	**0.04**
**Aromatic**	4.08 (2.38)	3.93 (2.54)	**0.04**
**Alkaline**	7.16 (4.78)	6.82 (4.45)	**0.03**
**Sulfuric**	1.71 (1.03)	1.55 (1.00)	**0.02**
**Acidic**	13.50 (7.00)	12.71 (7.78)	**0.04**
**Alcoholic**	3.99 (2.34)	3.78 (2.42)	0.05
**Small amino acids**	4.18 (2.67)	3.98 (2.54)	**0.03**
**Proline**	2.49 (1.49)	2.38 (1.51)	0.09
**Essential**	18.12 (10.98)	17.00 (11.35)	**0.04**
**Non-essential**	27.69 (15.25)	26.12 (16.49)	**0.04**

^a^Based on Mann-Whitney test; *IQR* Interquartile range

Using the univariable model, a significant association was found between T2D risk and branched-chain, alkaline, sulfuric, and essential amino acids in the fourth quartile. Accordingly, individuals in the fourth quartile had a 1.81- to 1.87-fold higher risk of developing new T2D than individuals in the lowest quartile (*P* < 0.05). In addition, after adjustment for several variables in different models, the risk of developing T2D increased with the higher intakes of amino acids but was not statistically significant for all amino acids. The risk of developing a new T2D was 2.70 (95% CI: 1.16–6.31), 2.68 (95% CI: 1.16–6.21), 2.98 (95% CI: 1.27–6.96), 2.45 (95% CI: 1.02–5.90), and 2.66 (95% CI: 1.13–6.25) times higher, for individuals in the fourth quartile of branched-chain, alkaline, sulfuric, alcoholic, and essential amino acids compared with those in the lowest quartile, respectively (Table 3).

**Table 3:** Crude and adjusted association of T2D with amino acid profile quartiles

Amino Acids	n case/control	Model I	Model II	Model III
		OR (95% CI)		
**Branched-chain**				
Q1	24/119	1.00	1.00	1.00
Q2	28/112	1.23 (0.67-2.26)	1.55 (0.79-3.03)	1.91 (0.92-3.97)
Q3	23/118	0.96 (0.51-1.80)	1.15 (0.57-2.33)	1.31 (0.58-2.94)
Q4	38/103	**1.82 (1.02-3.25)**	1.90 (0.98-3.70)	**2.70 (1.16-6.31)**
**Aromatic**				
Q1	24/118	1.00	1.00	1.00
Q2	27/114	1.16 (0.63-2.13)	1.30 (0.66-2.55)	1.59 (0.76-3.35)
Q3	26/115	1.11 (0.60-2.04)	1.30 (0.65-2.59)	1.47 (0.66-3.28)
Q4	36/105	1.68 (0.94-3.00)	1.67 (0.85-3.25)	2.36 (0.99-5.61)
**Alkaline**				
Q1	24/118	1.00	1.00	1.00
Q2	26/115	1.11 (0.60-2.04)	1.39 (0.70-2.73)	1.59 (0.76-3.32)
Q3	24/117	1.00 (0.54-1.87)	1.16 (0.58-2.32)	1.18 (0.52-2.66)
Q4	39/102	**1.87 (1.05-3.33)**	1.92 (0.99-3.73)	**2.68 (1.16-6.21)**
**Sulfuric**				
Q1	23/119	1.00	1.00	1.00
Q2	32/109	1.51 (0.83-2.75)	1.71 (0.88-3.31)	**2.22 (1.08-4.56**)
Q3	21/120	0.90 (0.47-1.72)	1.07 (0.52-2.19)	1.20 (0.53-2.74)
Q4	37/104	**1.84 (1.02-3.29**)	1.95 (0.99-3.83)	**2.98 (1.27-6.96)**
**Acidic**				
Q1	26/116	1.00	1.00	1.00
Q2	23/118	0.86 (0.46-1.61)	0.97 (0.49-1.92)	1.09 (0.51-2.31)
Q3	28/113	1.10 (0.61-2.00)	1.30 (0.66-2.54)	1.34 (0.61-2.94)
Q4	36/105	1.52 (0.86-2.70)	1.43 (0.74-2.77)	1.80 (0.74-4.35)
**Alcoholic**				
Q1	24/118	1.00	1.00	1.00
Q2	26/115	1.11 (0.60-2.04)	1.25 (0.63-2.45)	1.48 (0.70-3.11)
Q3	27/114	1.16 (0.63-2.13)	1.37 (0.69-2.72)	1.64 (0.74-3.64)
Q4	36/105	1.68 (0.94-3.00)	1.71 (0.87-3.34)	**2.45 (1.02-5.90**)
**Small amino acids**				
Q1	26/116	1.00	1.00	1.00
Q2	23/118	0.86 (0.46-1.61)	1.00 (0.50-1.99)	1.15 (0.54-2.45)
Q3	25/116	0.96 (0.52-1.76)	1.04 (0.53-2.04)	1.07 (0.48-2.39)
Q4	39/102	1.70 (0.97-2.99)	1.67 (0.87-3.20)	2.16 (0.94-4.97)
**Proline**				
Q1	26/116	1.00	1.00	1.00
Q2	26/115	1.00 (0.55-1.84)	1.13 (0.58-2.21)	1.22 (0.59-2.53)
Q3	29/112	1.15 (0.64-2.08)	1.14 (0.58-2.22)	1.26 (0.58-2.74)
Q4	32/109	1.30 (0.73-2.33)	1.37 (0.69-2.71)	1.67 (0.70-3.99)
**Essential**				
Q1	24/118	1.00	1.00	1.00
Q2	30/111	1.32 (0.73-2.41)	1.66 (0.85-3.22)	2.05 (0.99-4.24)
Q3	21/120	0.86 (0.45-1.62)	0.99 (0.48-2.01)	1.06 (0.46-2.42)
Q4	38/103	**1.81 (1.02-3.22)**	1.90 (0.97-3.73)	**2.66 (1.13-6.25)**
**Non-essential**				
Q1	25/118	1.00	1.00	1.00
Q2	25/115	1.02 (0.55-1.89)	1.16 (0.59-2.27)	1.36 (0.64-2.87)
Q3	28/113	1.16 (0.64-2.12)	1.43 (0.73-2.82)	1.58 (0.71-3.50)
Q4	35/106	1.55 (0.87-2.77)	1.45 (0.74-2.82)	1.89 (0.79-4.54)

Model I: unadjusted model; Model II: adjusted for age, sex, residency (urban, rural), education, SES (poor, moderate and high), *family history of diabetes,* BMI, WHtR, and physical activity (MET) (low, moderate and high) and daily energy intake; Model III: adjusted for model II and sleep duration (<6 h, 6-7 h and ≥8 h), dietary patterns (healthy or unhealthy), comorbidities (yes or no), systolic and diastolic blood pressure

Using a restricted cubic spline, there was no significant nonlinear association between dietary amino acid profiles and risk of T2D after adjustment for age, sex, place of residence, SES, education level, family history of diabetes, BMI, WHtR, physical activity, sleep duration, dietary patterns, comorbidities, systolic and diastolic blood pressure, and daily energy intake.

## Discussion

Amino acids have emerged as novel biomarkers for the risk of T2D. We conducted a nested case-control study in a cohort of 565 diabetic and healthy subjects. The results showed that the mean value of all dietary amino acid groups (except alcoholic and proline) was higher in patients with T2D than in the control group, and this result was statistically significant. A significant association between T2D risk and branched-chain, alkaline, sulfuric, and essential amino acids was found in the fourth quarter. Accordingly, individuals in the fourth quartile had a higher risk of developing new T2D than those in the lowest quartile. In addition, after adjustment for several variables in different models, the risk of T2D increased with increasing dietary amino acids but was not statistically significant for all amino acids. Overall, these amino acids may be useful new markers for identifying individuals at risk for T2D before symptoms become apparent. Insulin resistance may explain or mediate the relationship between these amino acids and the risk of T2D.

Previous studies have suggested several diabetes-related amino acids as potential biomarkers for insulin resistance and T2D. Higher levels of branched-chain amino acids have previously been associated with a higher risk of diabetes in European, Hispanic, African, and Asian populations [5, 13, 34, 35]. Furthermore, a large-scale Mendelian randomization analysis identified genetic instruments reflecting higher levels of circulating branched-chain species that are also associated with diabetes risk, suggesting a causal role of branched-chain amino acid metabolism in the development of diabetes [36].

In the study by Zheng et al. a meta-analysis of all cohorts comparing participants in the highest quintile with those in the lowest quintile of intake, the hazard ratios (HR) (95% confidence intervals) were 1.13 (95% CI: 1.07–1.19) for leucine, 1.13 (95% CI: 1.07–1.19) for isoleucine, and 1.11 (95% CI: 1.05–1.17) for valine. In a healthy subsample, higher dietary BCAA levels were significantly associated with higher plasma levels of these amino acids [1]. Wang et al. reported that individuals with the highest quartile of plasma BCAA concentrations had an approximately 3-fold higher risk of T2D than those with the lowest quartile [5]. A recent nested case-control study in the Framingham Offspring Study found that plasma BCAA levels are associated with fasting insulin levels and may predict future risk of diabetes, particularly in obese individuals and those with elevated fasting glucose levels [5].

A prospective population-based cohort study in Groningen, the Netherlands, showed that individuals with high circulating BCAA concentrations had a significantly higher risk of T2M. This association remained significant after adjustment for established risk factors such as age, sex, BMI, parental history of T2D, hypertension, alcohol consumption, HOMA-IR, and HOMA-β [37]. On the other hand, Franini et al. reported that 130 representative subjects from Bosnia who developed T2D after a follow-up period of 9.5 years had increased concentrations of leucine, isoleucine, and valine compared with 412 subjects who were free of T2D [8].

Several prospective studies have consistently reported an association between circulating BCAA concentrations and the development of T2D [13, 14, 37]. The meta-analysis by Ramzan et al. found a statistically significant positive association between BCAA concentrations and the development of T2D, with valine, leucine, and isoleucine [38]. Also, consistent with the results of Guasch-Ferre et al. a positive association between BCAAs and the incidence of T2D was demonstrated, with a pooled risk ratio (RR) for isoleucine, leucine, and valine of 1.36 (95% CI: 1.24–1.48), 1.36 (95% CI: 1.17–1.58), and 1.35 (95% CI: 1.19–1.53), respectively [7]. The study by Tai et al. showed a strong association between insulin resistance and branched-chain and aromatic amino acids and a combination of isoleucine, leucine, phenylalanine, and methionine in South Asian and Chinese men with relatively low body weight [39].

The study by Lu et al. showed that an increase in 6 essential amino acids (isoleucine, leucine, lysine, phenylalanine, tryptophan, and valine) was associated with a higher risk for prevalent and/or incident T2D. In addition, valine showed a positive predictive value for the risk of diabetes in this Chinese population [40]. In a nested case-control study of 429 Chinese adults, serum BCAA and aromatic amino acids (valine, leucine, isoleucine, phenylalanine, and tyrosine) were significantly and positively associated with T2D [13]. In a large-scale cross-sectional study among Japanese, individuals with T2D had higher BCAA levels than non-diabetics [41].

It is important to note that the effect size can be influenced by factors such as ethnicity, the variables that have been adjusted for, and the methods used for measuring BCAAs. In a study conducted by Lee (2016), adjusted OR were calculated for a one standard deviation (SD) increase in plasma BCAAs, which differs from our approach [42].

Alqudah et al. showed that plasma concentrations of leucine, lysine, phenylalanine, tryptophan, and glutamate were significantly increased in T2D patients compared with the control group and were positively related to poor glucose management [43].

Tryptophan is involved in the tryptophan-kynurenine and tryptophan-methoxy indole metabolic pathways, which leads to the production of some active metabolites, including kynurenine, kynurenic acid, and serotonin [43]. Any disturbance in these metabolic pathways is likely to be associated with the development of T2D [44].

Several previous studies reported a positive association between phenylalanine and the risk of T2D [6, 7, 45]. Among 1,150 participants in the Framingham Heart Study children cohort with normal fasting blood glucose, the adjusted HR for T2D risk per SD increase in phenylalanine was 1.35 (95% CI: 1.11–1.65), and a Mendelian randomization analysis found a similar relationship with the odds ratio (OR) of 1.60 (95% CI: 1.08–2.40) [45]. A case-control study reported that serum phenylalanine was independently associated with an increased risk of T2D [6]. A meta-analysis identified 27 cross-sectional studies and 19 prospective studies involving 8,000 subjects and found a 26% higher risk of T2D per study-specific SD [46].

In a case-cohort study, baseline lysine was found to be associated with a higher risk of T2D with an HR of 1.26 (95% CI: 1.06–1.51) per SD increment [47]. Another study also showed that an essential amino acid was elevated in people with T2D [43]. One study also found that cysteine was increased in T2D compared with controls and was associated with high HbA1c [43]. Histidine has also been positively associated with T2D, although the evidence is limited [7].

However, there are conflicting data on the role of BCAA as a mechanism for the observed association. Data from the Nagata et al. study suggest that high BCAA intake may be associated with a lower risk of diabetes [48].

In the present study, no significant association was found between the risk of T2D and aromatic, non-essential, acidic amino acids, small amino acids, and proline. Also, no association was found between glycine and the risk of T2D. This finding is consistent with the results of a cohort study in the United Kingdom, which reported that glycine was not associated with the incidence of T2D in South Asia [49]. An inverse association of glycine with T2D has been reported in previous European cohort studies [8, 10], whereas a Mendelian randomization analysis in a European population showed no association between genetic variants associated with glycine and T2D [14].

We found that glutamic acid and aspartic acid were not associated with an increased risk of T2D. In some studies, no association was found [5]. However, some other cohort studies have reported that high glutamine levels are associated with a lower risk of T2D [11, 50]. In addition, one study found that higher concentrations of total glutamate/glutamine were associated with insulin resistance and the development of diabetes in Chinese and Indian participants [39]. In one study, aspartate and glutamate were increased in individuals with T2D compared with healthy controls [43]. Existing evidence on the association between glutamate and the development of T2D is conflicting.

In this study, proline was not significantly associated with the risk of diabetes. In contrast, in other studies, proline was associated with an increased risk of developing T2D in all participants [46]. Another cross-sectional study showed that proline was strongly correlated with hemoglobin A1c and insulin-related variables such as C-peptide, insulin, and HOMA-IR [51].

Although not yet fully understood, there are several possible mechanisms underlying the association between amino acids and risk for T2D. First, type 2 diabetes begins with insulin resistance in peripheral tissues [13]. Some observations suggest that BCAA facilitates glucose uptake by skeletal muscle and liver and promotes glycogen synthesis in an insulin-independent manner through phosphatidylinositol 3-kinase (PI3-kinase) or protein kinase C (PKC) rather than the mTOR pathway [40, 52]. A recent study by Pedersen et al. showed that altered gut microbiota affects BCAA levels and may contribute to insulin resistance [53]. In addition, recent studies have indicated interactions between adipose tissue, BCAA metabolism, and glucose homeostasis. Increased BCAA may generate more catabolic intermediates propionyl CoA and succinyl CoA, leading to the accumulation of incompletely oxidized fatty acids and glucose, mitochondrial stress, impaired insulin action, and ultimately disruption of glucose homeostasis [13].

Our study has strengths and limitations. Strengths of our study include a prospective nested case-control design that allows measurement of exposures before T2D diagnosis, and the prospective nature of the study design minimizes the likelihood of recall and selection bias. Residual confounding is a common and unavoidable issue in observational studies. We sought to minimize the influence of potential confounders by controlling for potentially confounding variables, including important lifestyle risk factors. Racial homogeneity limited the generalizability of our findings to other ethnic groups. Replication of these results in another cohort is needed to increase the validity of our results.

## Conclusions

The results showed that the risk of developing T2D increased with increasing dietary amino acids, but was not statistically significant for all amino acids. The risk of developing a new T2D was higher for individuals in the fourth quartile of branched-chain amino acids, alkaline, sulfate, and essential amino acids than in the lower quartile. Therefore, in the detection of people who are at risk for T2D, these amino acids could be useful markers. Further studies in other populations should be conducted to investigate the association and to determine the positive and negative effects of related dietary amino acid patterns on chronic diseases.

### Supplementary Information

**Additional file 1.**

## Data Availability

The datasets used and/or analyzed during the current study are available from the corresponding author upon reasonable request.

## Abbreviations

BCAA: Branched-chain amino acids
BMI: Body mass index
BP: Blood pressure
CVD: Cardiovascular disease
FBS: Fasting blood glucose
FFQ: Food frequency questionnaire
HDL: High-density lipoprotein
HR: Hazard ratios
HTN: Hypertension
IQR: Interquartile range
LDL: Low-density lipoprotein
METs: Metabolic equivalents
OR: Odds ratio
PCA: Principal component analysis
PI3-kinase: Phosphatidylinositol 3-kinase
PKC: Protein kinase C
RaNCD: Ravansar Non-Communicable Disease
RR: Risk ratio
SD: Standard deviation
SES: Socioeconomic status
T2D: Type 2 diabetes
TG: Triglyceride
WC: Waist circumference
WHtR: Waist-to-height ratio
