# Supplementary material for: The association between dietary amino acid profile and the risk of type 2 diabetes: Ravansar non-communicable disease cohort study
Source: BMC Public Health. 2023 Nov 18;23:2284. doi: 10.1186/s12889-023-17210-5 (PMC10657569; doi:10.1186/s12889-023-17210-5)
Supplement: Supplementary file 1 — Additional file 1. [file 12889_2023_17210_MOESM1_ESM.docx]

**Distribution of dietary food groups by group (case & control)**

| **Dietary food groups (gr/day)** | **Case (n= 113)** | **Control (n= 452)** | **P-value^*^** |
| --- | --- | --- | --- |
|  | **Median (Interquartile range)** | |  |
| Whole grains | 24.60 (36.23) | 17.83 (28.75) | **0.005** |
| Refined grains | 441.64 (223.28) | 438.12 (243.56) | 0.758 |
| Legumes | 24.54 (30.70) | 23.49 (29.22) | 0.334 |
| Fresh fruits | 209.22 (209.20) | 177.41 (217.54) | **0.018** |
| Dried fruits | 17.35 (29.29) | 14.97 (23.87) | 0.055 |
| Fruit juices | 0 (3.78) | 0 (3.12) | 0.909 |
| Nuts | 6.69 (10.62) | 4.36 (8.62) | **0.002** |
| Red meats | 9.86 (21.69) | 9.86 (21.69) | 0.968 |
| Processed meats | 0 (1.27) | 0 (0.65) | 0.146 |
| Organ meats | 1.78 (3.61) | 1.80 (3.28) | 0.881 |
| Chicken meats | 0.98 (4.27) | 0 (3.45) | **0.034** |
| Poultry | 34.19 (47.01) | 25.64 (34.19) | 0.084 |
| Fish | 2.46 (8) | 2.08 (5.75) | 0.349 |
| Eggs | 11.83 (21.69) | 13.31 (21.69) | 0.454 |
| Saturated fats | 17.35 (37.19) | 19.25 (25.61) | 0.426 |
| Unsaturated fats | 4.27 (11.59) | 2.48 (10.26) | 0.113 |
| Leafy vegetables | 26.91 (29.15) | 21.19 (24.38) | **0.014** |
| Other vegetables | 433.04 (323.13) | 365.67 (298.14) | **0.029** |
| Dairy products | 308.05 (397.58) | 314.55 (422.10) | 0.968 |
| Drinks | 697.56 (485.43) | 690 (537.47) | 0.411 |
| Salts | 3 (3) | 3 (3) | 0.390 |
| Condiments | 20.72 (26.14) | 17.18 (21.43) | **0.007** |
| Sweets | 27.36 (37.52) | 36.84 (34.59) | **0.004** |
| Desserts | 12.82 (16.75) | 10.24 (15.30) | 0.849 |

**^*^Based on Mann-Whitney test**
